# Exome sequencing facilitates personalized treatment in developmental and epileptic encephalopathy patients: transforming current clinical practice in Indonesia

**DOI:** 10.1016/j.lansea.2025.100638

**Published:** 2025-07-19

**Authors:** Agung Triono, Kristy Iskandar, Auliya S.B. Sumpono, Tyas I. Hikmawan, Gunadi, Elisabeth S. Herini

**Affiliations:** a Department of Child Health, Faculty of Medicine, Public Health, and Nursing, Universitas Gadjah Mada/Dr. Sardjito Hospital, Yogyakarta, Indonesia; b Department of Child Health, Faculty of Medicine, Public Health, and Nursing, Universitas Gadjah Mada/UGM Academic Hospital, Yogyakarta, Indonesia; c Department of Anatomical Pathology, Faculty of Medicine, Public Health, and Nursing, Universitas Gadjah Mada/Dr. Sardjito Hospital, Yogyakarta, Indonesia; d Department of Tropical Biology, Faculty of Biology, Universitas Gadjah Mada, Yogyakarta, Indonesia; e Pediatric Surgery Division, Department of Surgery, Genetic Working Group/Translational Research Unit, Faculty of Medicine, Public Health, and Nursing, Universitas Gadjah Mada/Dr. Sardjito Hospital, Yogyakarta, Indonesia; f Rare Disease Hub, Dr. Sardjito Hospital, Biomedical and Genome Science Initiative (BGSi), Ministry of Health, Indonesia

Developmental epilepsy and encephalopathies (DEEs) are among the most severe groups of epilepsies, characterized by frequent, often drug-resistant seizures, distinct epileptiform abnormalities on electroencephalography (EEG), and developmental regression or cognitive impairments. In many cases, the underlying etiology contributes directly to developmental impairment.^1^, ^2^, ^3^, ^4^

In Indonesia, the selection of antiseizure medications (ASMs) for DEEs is largely based on an empirical approach guided by the apparent seizure type, such as generalized, focal, or absence seizure. However, this method may not align with the underlying pathogenetic mechanisms and, in some cases, may exacerbate seizure activity. Distinguishing whether seizure worsening is due to inappropriate medication or reflects the inherent pharmacoresistance of DEEs is often challenging. Furthermore, access to first-line treatments for specific DEE remains limited across the Southeast Asia region.^5^

Clarifying an etiology-specific diagnosis can significantly inform treatment decisions in patients with DEEs. Although genetic causes have been implicated in up to 50% of DEE cases—spanning more than 900 genes^4^—most children in low- and middle-income countries (LMICs) continue to be managed empirically, without molecular diagnosis and often without access to disease-modifying options. In Indonesia, this diagnostic gap persisted due to the limited availability and clinical integration of genomic testing.

This gap is particularly concerning for neurometabolic disorders—a treatable subset of DEEs. Indonesia’s national newborn screening program does not currently include testing for inherited metabolic disorders.^6^ Biochemical diagnostics, such as plasma amino acid analysis, acylcarnitine profiling, or urine organic acid testing, are rarely available in routine clinical settings. As a result, many cases of metabolic epilepsies go undiagnosed. Other genetic causes—such as channelopathies and non-structural epileptic encephalopathies—are similarly under-recognized due to the lack of access to comprehensive genomic diagnostics.

Our recent study provides compelling evidence that exome sequencing is both feasible and clinically valuable in an LMIC setting. Among 57 patients, we identified pathogenic or likely pathogenic variants in 17 (29.8%), and variants of uncertain significance (VUS) in 9 (15.7%), based on the American College of Medical Genetics and Genomics guidelines.^7^ In total, 26 of 57 patients (45.6%) had a possible genetic etiology, with ten (17.5%) having diagnoses with direct therapeutic implications. These included targeted treatment of underlying biochemical abnormalities (e.g., vitamin or metabolic enzymatic-diet management), rational modification of ASMs based on molecular diagnosis, surveillance for disease-related complications, and potential enrollment in gene-specific clinical trials. Notably, four of these ten cases were neurometabolic disorders. Beyond clinical management, these findings offered diagnostic closure for families navigating complex and prolonged diagnostic journeys (Fig. 1).^8^

**Fig 1:**
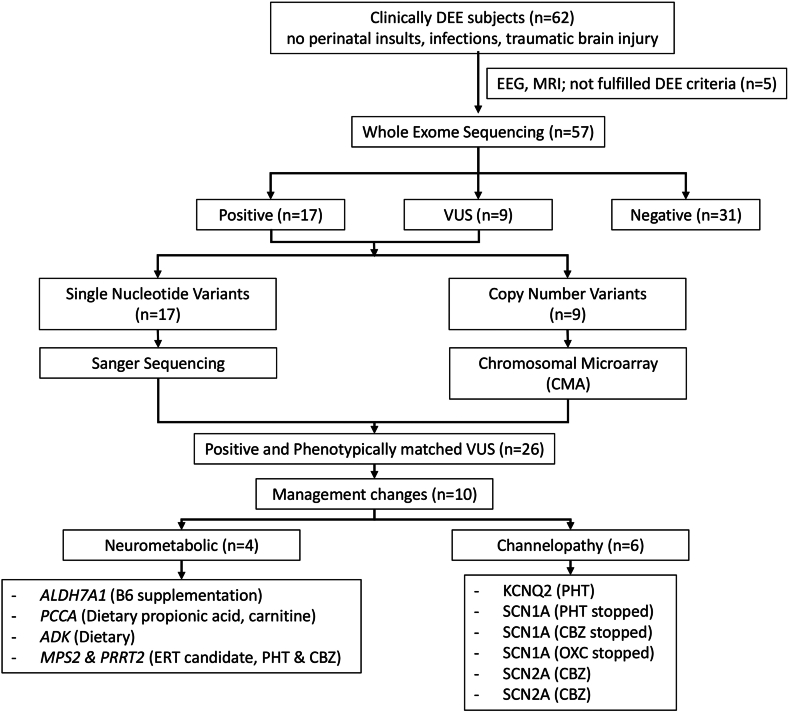
**Workflow strategy and management changes from the genetic testing results**.

While exome sequencing has become a routine part of care for DEEs in high-income countries—now moving toward genome sequencing—its use in LMICs remains limited and often questioned. Our finding demonstrates that not only is exome sequencing feasible in such settings, but its implementation can also be transformative. It delivers definitive diagnoses, refines clinical decision-making, informs prognosis, and enables genetic counselling.

Despite real challenges—cost, infrastructure, and workforce limitations—these barriers are not insurmountable. With strategic national investments, community-supported funding, collaborative efforts across academic and clinical institutions, and selective out-of-pocket funding, exome sequencing can be implemented selectively for high-yield indications such as early-onset epilepsies of unknown cause. Compared to other genetic modalities such as metabolic panels, chromosomal microarray, or targeted panels, exome sequencing offers broader coverage and may be more cost-effective.^9^

Our study, supported by national research funding and multidisciplinary collaboration, illustrates a scalable model. At approximately USD 350 per test, the cost of exome sequencing is modest compared to the typical eight-year diagnostic odyssey and the cumulative burden of untreated disease. Moreover, non-stepwise diagnostic approaches may be more economical in the long term.^9^^,^^10^

It is time to redefine essential care. Exome sequencing should be considered a first-line investigation for infants with unexplained, treatment-resistant epilepsy. Early molecular diagnosis not only improves therapeutic decision-making but also opens access to emerging gene-based treatments and international trials as precision medicine evolves.

Indonesia’s experience demonstrates that genomic medicine is not a distant ideal-it is a practical and impactful tool. For children with DEEs, a timely and accurate diagnosis can mean the difference between lifelong disability and meaningful intervention. This evidence should catalyze a shift in health policy: exome sequencing must be recognized not as a luxury, but as a standard of care in pediatric neurology.^11^

## Contributors

Agung Triono-literature search, study design, data collection, data analysis, data interpretation.

Kristy Iskandar-literature search, study design, data collection, data analysis, data interpretation and writing.

Auliya SB Sumpono-data collection, data analysis, data interpretation and writing.

Tyas I Hikmawan-data analysis, data interpretation, bioinformatics.

Gunadi-data analysis, data interpretation and writing.

Elisabeth Siti Herini-data analysis, data interpretation and writing.

## Declaration of interests

None declared.
