# Supplementary material for: Exome sequencing facilitates personalized treatment in developmental and epileptic encephalopathy patients: transforming current clinical practice in Indonesia
Source: Lancet Reg Health Southeast Asia. 2025 Jul 19;39:100638. doi: 10.1016/j.lansea.2025.100638 (PMC12302292; doi:10.1016/j.lansea.2025.100638)
Supplement: Supplementary Table 1 [file mmc1.docx]

| **No** | **Results**  **Supplementary Table 1.** Variant-Level Findings and Management Changes | | | | | **Population Data** | | **Predicted Consequence**  **/Location** | | **Segregation Data** | **Computation and Functional Data** | **Previously Reported Variant Data** | **Disease Association** | | **Validation** | | **Variant Classification** | | **Management Changes** | |
| --- | --- | --- | --- | --- | --- | --- | --- | --- | --- | --- | --- | --- | --- | --- | --- | --- | --- | --- | --- | --- |
|  | **Interpretation** | **Diagnosis** | **Gene** | **Variant** |  | |  | |  | |  |  |  |  | |  | |  | |  |
| 1 | POSITIVE | Mucopolysaccharidosis II  (MIM #309900)  PRRT2-related disorder  (MIM #614386) | MPS2  PRRT2 | NM_000202.8:c.1620dup (p.Gly541ArgfsTer124)    NM_145239.3:c.919C>A (p.Gln307Lys) | The variant is not observed in the gnomAD v4.1.0 dataset. | | Frameshift: predicted to result in a loss or disruption of normal protein function through protein truncation.  Multiple pathogenic variants are reported in the predicted truncated region.  Missense variant. The majority of the known disease-causing variants of this gene are variants expected to  result in premature termination of the protein. | | None | | In silico tool predictions suggest damaging effect of the variant on gene or gene product [3Cnet: 0.99 (> 0.75,  sensitivity 0.96 and precision 0.92)]. | The variant has been reported as of uncertain significance (ClinVar ID: VCV002435264). Different missense  changes at the same codon have been reported as of uncertain significance (ClinVar ID: VCV001433481) | Mucopolysaccharidosis II (OMIM: 309900)  PRRT2-related disorder (OMIM: 614386) | Not performed as the variant was considered high-quality | | Likely pathogenic  VUS | | CBZ; no access to ERT 🡪 no changes on seizures yet | |  |
| 2 | INCONCLUSIVE | DEE 11  (MIM #613721) | SCN2A | NM_001040142.2:c787G>C (p.Ala263Pro) | The variant is not observed in the gnomAD v4.1.0 dataset. | | Missense variant | | None | | In silico tool predictions suggest damaging effect of the variant on gene or gene product [REVEL: 0.96 (>=0.6,  sensitivity 0.68 and specificity 0.92); 3Cnet: 0.99 (> 0.75, sensitivity 0.96 and precision 0.92)]. | Different missense changes at the same codon (p.Ala263Glu, p.Ala263Gly, p.Ala263Thr, p.Ala263Val) have  been reported as pathogenic/likely pathogenic with strong evidence (ClinVar ID: VCV000029888,  VCV000520403, VCV001070304, VCV002939257 / PMID: 20956790, 23935176 / 3bilion dataset). | SCN2A-related disorder (OMIM: 182390) | Not performed as the variant was considered high-quality | | VUS | | CBZ 🡪 seizures stopped | |  |
| 3 | INCONCLUSIVE | Epilepsy, pyridoxine-dependent (OMIM: 266100) | ALDH7A1 | Genomic Position: 5-125891655-T-C (GRCh37)  cDNA: NM_001182.5:c.1061A>G Protein: NP_001173.2:p.Tyr354Cys Zygosity: Heterozygous Inheritance: Unknown | The variant is observed at an extremely low frequency in the gnomAD v2.1.1 dataset (total allele frequency: 0.001%). | | Missense variant | | None | | In silico tool predictions suggest damaging effect of the variant on gene or gene product [REVEL: 0.75 (>=0.6, sensitivity 0.68 and specificity 0.92); 3Cnet: 0.85 (>=0.6, sensitivity 0.72 and precision 0.9)]. | Same nucleotide change resulting in same amino acid change has been previously reported as pathogenic/likely pathogenic with strong evidence (ClinVar ID: VCV001074455 / PMID: 22371912). The variant has been reported to be in trans with a pathogenic variant as either compound heterozygous or homozygous in at least 4 similarly affected unrelated individuals (PMID: 31737911, 33822359). A different missense change at the same codon (p.Tyr354Ser) has been reported to be associated with ALDH7A1 related disorder (ClinVar ID: VCV002500759). | Epilepsy, pyridoxine-dependent (OMIM: 266100) | Not performed as the variant was considered high-quality | | Pathogenic | | B6, lysin restriction, arginine supplementation 🡪 seizure stop | |  |
|  |  |  | ALDH7A1 | Genomic Position: 5-125882067-C-A (GRCh37)  cDNA: NM_001182.5:c.1514G>T Protein: NP_001173.2:p.Gly505Val Zygosity: Heterozygous Inheritance: Unknown | The variant is not observed in the gnomAD v2.1.1 dataset. | | Missense variant | | None | | In silico tool predictions suggest damaging effect of the variant on gene or gene product [REVEL: 0.91 (>=0.6, sensitivity 0.68 and specificity 0.92); 3Cnet: 0.96 (>=0.6, sensitivity 0.72 and precision 0.9)]. | A different missense change at the same codon (p.Gly505Arg) has been reported as pathogenic/likely pathogenic with strong evidence (ClinVar ID: VCV000204852 / PMID: 19128417). | Epilepsy, pyridoxine-dependent (OMIM: 266100) | Not performed as the variant was considered high-quality | | VUS | |  | |  |
|  |  |  | ALDH7A1 | Genomic Position: 5-125912795-A-G (GRCh37)  cDNA: NM_001182.5:c.626T>C Protein: NP_001173.2:p.Met209Thr Zygosity: Heterozygous Inheritance: Unknown | The variant is not observed in the gnomAD v2.1.1 dataset. | | Missense variant | | None | | In silico tool predictions suggest damaging effect of the variant on gene or gene product [REVEL: 0.69 (>=0.6, sensitivity 0.68 and specificity 0.92); 3Cnet: 0.99 (>=0.6, sensitivity 0.72 and precision 0.9)]. | None | Epilepsy, pyridoxine-dependent (OMIM: 266100) | Not performed as the variant was considered high-quality | | VUS | |  | |  |
| 4 | POSITIVE Variant was observed: 1. PCCA (NM_000282.4:c.183+1G>A (p.?) 2. NM_000282.4:c.1937del (p.Leu646Ter) | Propionicacidemia (OMIM: 606054) | PCCA | Genomic Position: 13-100755215-G-A (GRCh37) DNA: NM_000282.4:c.183+1G>A Protein: NP_000273.2:p.? Zygosity: Heterozygous Inheritance: Unknown | The variant is observed at an extremely low frequency in the gnomAD v2.1.1 dataset (total allele frequency: 0.001%). | | Canonical splice site: predicted to alter splicing and result in a loss or disruption of normal protein function. Multiple pathogenic loss-of-function variants are reported downstream of the variant. | | None | | None | The variant has been reported to be associated with PCCA related disorder (ClinVar ID: VCV001066791). | Propionicacidemia (OMIM: 606054) | Not performed as the variant was considered high-quality | | Pathogenic | | Dietary propionic acid, carnitine 🡪 seizure controlled | |  |
|  |  |  | PCCA | Genomic Position: 13-101167716-AT-A (GRCh37) DNA: NM_000282.4:c.1937del Protein: NP_000273.2:p.Leu646Ter Zygosity: Heterozygous Inheritance: Unknown | The variant is not observed in the gnomAD v2.1.1 dataset. | | Stop-gained (nonsense): predicted to result in a loss or disruption of normal protein function through nonsense-mediated decay (NMD) or protein truncation. Multiple pathogenic variants are reported downstream of the variant. | | None | | None | None | Propionicacidemia (OMIM: 606054) | Not performed as the variant was considered high-quality | | Likely Pathogenic | |  | |  |
| 5 | INCONCLUSIVE | Hypermethioninemia due to adenosine kinase deficiency (OMIM: 614300) | ADK | Genomic Position: 10-76153999-T-C (GRCh37) cDNA: NM_006721.4:c.374T>C Protein: NP_006712.2:p.Val125Ala Zygosity: Homozygous Inheritance: Unknown | The variant is not observed in the gnomAD v2.1.1 dataset. | | Missense changes are a common disease-causing mechanism. | | None | | In silico tool predictions suggest damaging effect of the variant on gene or gene product (REVEL: 0.90; 3Cnet: 0.12). | None | Hypermethioninemia due to adenosine kinase deficiency (OMIM: 614300) | Not performed as the variant was considered high-quality | | VUS | | Dietary 🡪 seizure stopped | |  |
| 6 | POSITIVE KCNQ2 NM_172107.4:c.868G>A (p.Gly290Ser) | Epileptic encephalopathy, early infantile, 7 (OMIM: 613720) | KCNQ2 | Genomic Position: 20-62071010-C-T (GRCh37) cDNA: NM_172107.4:c.868G>A Protein: NP_742105.1:p.Gly290Ser Zygosity: Heterozygous Inheritance: Unknown | The variant is not observed in the gnomAD v2.1.1 dataset. | | Missense changes are a common disease-causing mechanism. | | None | | In silico tool predictions suggest damaging effect of the variant on gene or gene product (REVEL: 0.96; 3Cnet: 0.94). | Same nucleotide change resulting in same amino acid change (ClinVar ID: VCV000369768) and different missense changes at the same codon (p.Gly290Asp, p.Gly290Val / ClinVar ID: VCV000039762, VCV000523563) have been previously reported as pathogenic/likely pathogenic with strong evidence. | Epileptic encephalopathy, early infantile, 7 (OMIM: 613720) | Not performed as the variant was considered high-quality | | Pathogenic | | PHT 🡪 seizures frequency decreased | |  |
| 7 | POSITIVE | Dravet syndrome (OMIM: 607208) | SCN1A | Genomic Position: 2-166859265-T-C (GRCh37) DNA: NM_001165963.4:c.4003-2A>G Protein: NP_001159435.1:p.? Zygosity: Heterozygous Inheritance: Unknown | The variant is not observed in the gnomAD v2.1.1 dataset. | | Canonical splice site: predicted to alter splicing and result in a loss or disruption of normal protein function. Multiple pathogenic loss-of-function variants are reported downstream of the variant. | | None | | None | None | Dravet syndrome (OMIM: 607208) | Not performed as the variant was considered high-quality | | Likely Pathogenic | | PHT stopped 🡪 seizures frequency decreased | |  |
| 8 | INCONCLUSIVE | SCN1A-related disorder (OMIM: 182389) | SCN1A | Genomic Position: 2-166929894-G-T (GRCh37)  cDNA: NM_001165963.4:c.238C>A Protein: NP_001159435.1:p.Leu80Met Zygosity: Heterozygous Inheritance: Unknown | The variant is not observed in the gnomAD v2.1.1 dataset. | | Missense variant | | None | | In silico tool predictions suggest damaging effect of the variant on gene or gene product [3Cnet: 0.79 (>=0.6, sensitivity 0.72 and precision 0.9)]. | None | SCN1A-related disorder (OMIM: 182389) | Not performed as the variant was considered high-quality | | VUS | | CBZ stopped 🡪 seizures frequency decreased | |  |
| 9 | POSITIVE | Dravet syndrome (OMIM: 607208) | TTC21B, SCN1A, SCN9A | Genomic Position: 2:(?_166731265)_(167168266_?)[DEL] (GRCh37) Cytogenetic band: 2q24.3 (minimum size: 437Kb) Type: Deletion Zygosity: Heterozygous Inheritance: Unknown | The variant is not observed in the gnomAD v2.1.1 dataset. | | None | | There are multiple similarly affected individuals reported with similar likely pathogenic copy-number-loss overlapping this region (PMID: 25524840, 27113213). | | None | Other pathogenic variants have been reported in this region. | Dravet syndrome (OMIM: 607208) | WGS | | Pathogenic | | OXC stopped 🡪 seizures frequency decreased | |  |
| 10 | INCONCLUSIVE | Developmental and epileptic encephalopathy 11 (OMIM: 613721) | SCN2A | Genomic Position: 2-166166890-T-C (GRCh37)  cDNA: NM_001040142.2:c.755T>C Protein: NP_001035232.1:p.Met252Thr Zygosity: Heterozygous Inheritance: Unknown | The variant is not observed in the gnomAD v2.1.1 dataset. | | Missense variant | | None | | In silico tool predictions suggest damaging effect of the variant on gene or gene product [REVEL: 0.95 (>=0.6, sensitivity 0.68 and specificity 0.92); 3Cnet: 0.95 (>=0.6, sensitivity 0.72 and precision 0.9)]. | Different missense changes at the same codon (p.Met252Ile, p.Met252Val) have been reported to be associated with SCN2A related disorder (ClinVar ID: VCV000029889 / PMID: 20371507, 34894057). However the evidence of pathogenicity is insufficient at this time. | Developmental and epileptic encephalopathy 11 (OMIM: 613721) | Not performed as the variant was considered high-quality | | VUS | | CBZ 🡪 seizures stopped | |  |
| 11 | POSITIVE Variant was observed 1: 4-107258117-C-A (GRCh37) | Leukodystrophy, hypomyelinating, 3 (OMIM: 260600) | AIMP1 | Genomic Position: 4-107258117-C-A (GRCh37) DNA: NM_001142416.2:c.695C>A Protein: NP_001135888.2:p.Ser232Ter Zygosity: Homozygous Inheritance: Unknown | The variant is not observed in the gnomAD v2.1.1 dataset. | | Stop-gained (nonsense): predicted to result in a loss or disruption of normal protein function through nonsense-mediated decay (NMD) or protein truncation. Multiple pathogenic variants are reported downstream of the variant. | | Regardless of the mechanism, a variant called homozygous is by default in trans. | | None | None | Leukodystrophy, hypomyelinating, 3 (OMIM: 260600) | Not performed as the variant was considered high-quality | | Pathogenic | |  | |  |
| 12 | POSITIVE ARX NM_139058.3:c.1206del (NP_620689.1:p.Pro403ArgfsTer60) | Lissencephaly, X-linked 2 | ARX | Genomic Position: X-25025469-GC-G cDNA: NM_139058.3:c.1206del Protein: (NP_620689.1:p.Pro403ArgfsTer60) Zygosity: Heterozygous Inheritance: Unknown | It is absent from the gnomAD v2.1.1 dataset. | | The deletion creates a frameshift variant, which is expected to cause a loss of normal protein function via nonsense-mediated mRNA decay. | | None | | None | None | Lissencephaly, X-linked 2 | Not performed as the variant was considered high-quality | | Likely Pathogenic | |  | |  |
| 13 | INCONCLUSIVE (POSITIVE Variant was observed 1: Variant 1 X-25031039-C-T (GRCh37) | Developmental and epileptic encephalopathy 1 (OMIM: 308350) | ARX | Genomic Position: X-25031039-C-T (GRCh37) DNA: NM_139058.3:c.1073G>A Protein: NP_620689.1:p.Arg358Lys Zygosity: Hemizygous Inheritance: Unknown | The variant is not observed in the gnomAD v2.1.1 dataset. | | Missense variant | | None | | In silico tool predictions suggest damaging effect of the variant on gene or gene product REVEL: 0.75 (0.6, sensitivity 0.68 and specificity 0.92). | Different missense changes at the same codon (p.Arg358Ser, p.Arg358Trp) have been reported to be associated with ARX related disorder (PMID: 21416597, 21496008). However the evidence of pathogenicity is insufficient at this time. | Developmental and epileptic encephalopathy 1 (OMIM: 308350) | Not performed as the variant was considered high-quality | | VUS | |  | |  |
| 14 | INCONCLUSIVE | Neurodevelopmental disorder with hypotonia, language delay, and skeletal defects with or without seizures (OMIM: 620029) | CACNA1C | Genomic Position: 12-2675692-C-T (GRCh37) DNA: NM_000719.7:c.1613C>T Protein: NP_000710.5:p.Thr538Met Zygosity: Heterozygous Inheritance: Unknown | The variant is not observed in the gnomAD v2.1.1 dataset. | | Missense changes are a common disease-causing mechanism. | | None | | In silico tool predictions suggest damaging effect of the variant on gene or gene product REVEL: 0.91 (0.6, sensitivity 0.68 and specificity 0.92); 3Cnet: 0.65 (0.6, sensitivity 0.72 and precision 0,9)] | None | Neurodevelopmental disorder with hypotonia, language delay, and skeletal defects with or without seizures (OMIM: 620029) | Not performed as the variant was considered high-quality | | VUS | |  | |  |
| 15 | POSITIVE | Intellectual developmental disorder, X-linked 1 (OMIM: 309530) | IQSEC2 | Genomic Position: X-53265678-C-T (GRCh37)  cDNA: NM_001111125.3:c.3278-1G>A Protein: NP_001104595.1:p.? Zygosity: Hemizygous Inheritance: Unknown | The variant is not observed in the gnomAD v2.1.1 dataset. | | Canonical splice site: predicted to alter splicing and result in a loss or disruption of normal protein function. Multiple pathogenic loss-of-function variants are reported downstream of the variant. | | None | | In silico tools predict the variant to alter splicing and produce an abnormal transcript [Splice AI: 0.94 (spliceogenicity >=0.2, non-spliceogenicity <0.1)]. | None | Intellectual developmental disorder, X-linked 1 (OMIM: 309530) | Not performed as the variant was considered high-quality | | Likely pathogenic | |  | |  |
| 16 | POSITIVE | Arboleda-Tham syndrome (OMIM: 616268) | KAT6A | Genomic Position: 8-41792353-G-A (GRCh37)  cDNA: NM_006766.5:c.3385C>T Protein: NP_006757.2:p.Arg1129Ter Zygosity: Heterozygous Inheritance: Unknown | The variant is not observed in the gnomAD v2.1.1 dataset. | | Stop-gained (nonsense): predicted to result in a loss or disruption of normal protein function through protein truncation. The predicted truncated protein may be shortened by more than 10%. | | None | | None | The variant has been reported at least twice as pathogenic with clinical assertions and evidence for the classification (ClinVar ID: VCV000180229 / PMID: 25728775 / 3billion dataset). | Arboleda-Tham syndrome (OMIM: 616268) | Not performed as the variant was considered high-quality | | Pathogenic | |  | |  |
| 17 | POSITIVE | Neurofibromatosis,type 1 | NF1 | Genomic Position: 17-29576097-TC-T cDNA: NM_001042492.3:c.4076del Protein: (NP_001035957.1:p.Pro1359LeufsTer19) Zygosity: Heterozygous Inheritance: Unknown Mother's Variant: Negative | It is absent from the gnomAD v2.1.1 dataset. | | The deletion creates a frameshift variant, which is expected to cause a loss of normal protein function via nonsense-mediated mRNA decay. | | None | | None | This variant has been reported as pathogenic more than twice (ClinVar ID: VCV000431632, PMID:9003501), along with assertion criteria based on the ACMG guidelines. | Neurofibromatosis,type 1 | Not performed as the variant was considered high-quality | | Pathogenic | |  | |  |
| 18 | INCONCLUSIVE (POSITIVE Variant was observed 1 2-46840904-G-C GRCh37: PIGF NM_002643.4:c.249C>G (p.Cys83Trp) | Onychodystrophy, osteodystrophy, impaired intellectual development, and seizures syndrome (OMIM: 619356) | PIGF | Genomic Position: 2-46840904-G-C (GRCh37) DNA: NM_002643.4:c.249C>G Protein: NP_002634.1:p.Cys83Trp Zygosity: Homozygous Inheritance: Unknown | The variant is observed at an extremely low frequency in the gnomAD v2.1.1 dataset (total allele frequency: 0.008%). | | Missense variant | | None | | None | None | Onychodystrophy, osteodystrophy, impaired intellectual development, and seizures syndrome (OMIM: 619356) | Not performed as the variant was considered high-quality | | VUS | |  | |  |
| 19 | POSITIVE | Tuberous sclerosis-2 | TSC2 | Genomic Position: 16-2137898-C-T cDNA: NM_000548.5:c.5024C>T Protein: (NP_000539.2:p.Pro1675Leu) Zygosity: Heterozygous Inheritance: Unknown | The variant is not observed in the gnomAD v2.1.1 dataset. | | None | | The same variant was observed in multiple affected individuals with a consistent phenotype from unrelated families (PMID: 9302281, 11520734, 12111193). | | Functional assays showed thatthe variant had strong level of impact on gene/protein function (PMID: 22903760, 11290735). In silico prediction tools and conservation analysis predicted that this variant was probably damaging to the protein structure/function (REVEL: 0.965>=0.6, 3CNET: 0.939>=0.75). | Amino acid change identical to known pathogenic variant has been previously reported with established evidence (ClinVar ID: VCV000012393). Different pathogenic amino acid change has been reported with sufficient evidence at the same codon (ClinVarID: VCV000535873). | Tuberous sclerosis-2 | Not performed as the variant was considered high-quality | | Pathogenic | |  | |  |
| 20 | INCONCLUSIVE | Intellectual developmental disorder, autosomal dominant 22 (OMIM: 612337) | ZBTB18 | Genomic Position: 1-244218401-C-T (GRCh37) DNA: NM_205768.3:c.1325C>T Protein: NP_991331.1:p.Ser442Leu Zygosity: Heterozygous Inheritance: Unknown | The variant is not observed in the gnomAD v2.1.1 dataset. | | Missense changes are a common disease-causing mechanism. | | None | | In silico tool predictions suggest damaging effect of the variant on gene or gene product 3Cnet: 0.91 (0.6, sensitivity 0.72 and precision 0.9) | None | Intellectual developmental disorder, autosomal dominant 22 (OMIM: 612337) | Not performed as the variant was considered high-quality | | VUS | |  | |  |
| 21 | POSITIVE | 15q11.2q13.1 Deletion (OMIM: 176270, GeneReviews: NBK1144, ClinGen: ISCA-37478) | GOLGA6L2, MKRN3, MAGEL2, NDN, NPAP1, SNURF, SNRPN, UBE3A, ATP10A, GABRB3, GABRA5, GABRG3, OCA2, HERC2 | Genomic Position: 15:(?_23684892)_(28544662_?)[DEL] (GRCh37) Cytogenetic band: 15q11.2q13.1 (minimum size: 4.86Mb) Type: Loss Zygosity: Heterozygous Inheritance: Unknown | The variant is not observed in the gnomAD v2.1.1 dataset. | | None | | None | | None | None | Deletion at this genomic region is associated with autosomal dominant 'Prader-Willi syndrome (OMIM: 176270, GeneReviews: NBK1330)' and 'Angelman syndrome (GeneReviews: NBK1144). | Not performed as the variant was considered high-quality | | Pathogenic | |  | |  |
| 22 | POSITIVE | 15q11.2q13.1 Deletion (ClinGen: ISCA-37404) | TUBGCP5, CYFIP1, NIPA2, NIPA1, GOLGA8S, GOLGA6L2, MKRN3, MAGEL2, NDN, NPAP1, SNURF, SNRPN, UBE3A, ATP10A, GABRB3, GABRA5, GABRG3, OCA2, HERC2 | Genomic Position: 15:(?_22833525)_(28544662_?)[DEL] (GRCh37) Cytogenetic band: 15q11.2q13.1(minimum size:5.7Mb) Type: Loss Zygosity: Heterozygous Inheritance: Unknown | The variant is not observed in the gnomAD v2.1.1 dataset. | | None | | None | | None | None | Deletion at this genomic region is associated with autosomal dominant 'Prader-Willi syndrome (OMIM: 176270, GeneReviews: NBK1330)' and 'Angelman syndrome (OMIM: 105830, GeneReviews: NBK1144). | Not performed as the variant was considered high-quality | | Pathogenic | |  | |  |
| 23 | POSITIVE | Williams-Beuren syndrome (OMIM: 194050) | ZNF107, ZNF138, ZNF273, ZNF117, ERV3-1, ZNF92, VKORC1L1, GUSB, ASL, CRCP, TPST1, KCTD7, RABGEF1, TMEM248, SBDS, TYW1, AUTS2, WBSCR17, GALNT17, CALN1, TYW1B, POM121, TRIM74, SPDYE8, NSUN5, TRIM50, FKBP6, FZD9, BAZ1B, BCL7B, TBL2, MLXIPL, VPS37D, DNAJC30, WBSCR22, BUD23, STX1A, ABHD11, CLDN3, CLDN4, METTL27, WBSCR27, TMEM270, ELN, LIMK1, EIF4H, LAT2, RFC2, CLIP2, GTF2IRD1, GTF2I, NCF1, GTF2IRD2, GATSL2, GATSL1, CASTOR2, RCC1L, WBSCR16, GTF2IRD2B, SPDYE13, SPDYE14, TRIM73, POM121C, SPDYE5, HIP1, CCL26, CCL24, RHBDD2, POR, TMEM120A, STYXL1, MDH2, SRRM3, HSPB1, YWHAG, SSC4D, ZP3, DTX2, UPK3B, SPDYE16, POMZP3, SPDYE9, SPDYE17, SPDYE18, CCDC146, FGL2, GSAP, PTPN12, RSBN1L, TMEM60, PHTF2, MAGI2. | Genomic Position: 7:(?_64126661)_(77975360_?) [DEL] (GRCh37) Cytogenetic band: 7q11.21q21.11 (minimum size: 13.9Mb) Type: Loss Zygosity: Heterozygous Inheritance: Unknown | The variant is not observed in the gnomAD SVs v2.1.1 dataset. | | None | | There are multiple similarly affected individuals reported with similar likely pathogenic copy-number-loss overlapping this region (PMID: 20301427 ClinVar ID: VCV000814990.1 Decipher: 395348). | | None | Other pathogenic variants have been reported in this region. | Williams-Beuren syndrome (OMIM: 194050) | Not performed as the variant was considered high-quality | | Pathogenic | |  | |  |
| 24 | POSITIVE | Angelman syndrome (OMIM: 105830) and Prader-Willi syndrome (OMIM: 176270) | GOLGA6L2, MKRN3, MAGEL2, NDN, PWRN4, PWRN2, PWRN3, PWRN1, NPAP1, SNHG14, SNRPN, SNURF, SNORD107, PWARSN, PWAR5, SNORD64, SNORD108, PWAR6, SNORD109A, IPW, PWAR1, PWAR4, SNORD109B, UBE3A, ATP10A, GABRB3, GABRA5, GABRG3, OCA2, HERC2. | Genomic Position: Genomic Position: NC_000015.9:g.(?_23684892)_(28544662_?)del (GRCh37)  Cytogenetic band: 15q11.2q13.1 (minimum size: 4.8 Mb)  Type: Deletion Zygosity: Heterozygous Inheritance: Unknown | The variant is not observed in the gnomAD SVs v2.1.1 dataset. | | heterozygous deletion NC_000015.9:g.(?_23684892)_(28544662_?) at 15q11.2q13.1 spans across 30 genes | | None | | None | There are multiple similarly affected individuals reported with similar likely pathogenic copy-number-loss overlapping this region (PMID: 35956251; Decipher: 370084; GeneReviews: NBK1144, NBK1330) | This region spans well-known imprinted gene clusters associated with Angelman syndrome (OMIM: 105830) and Prader-Willi syndrome (OMIM: 176270) | Not performed as the variant was considered high-quality | | Pathogenic | |  | |  |
| 25 | POSITIVE | Autosomal dominant 'Miller-Dieker lissencephaly syndrome (OMIM: 247200) | DOC2B, RPH3AL, LOC100506388, LIAT1, RFLNB, VPS53, TLCD3A, GEMIN4, GLOD4, MRM3, NXN, TIMM22, ABR, BHLHA9, TRARG1, YWHAE, CRK, MYO1C, INPP5K, PITPNA, SLC43A2, SCARF1, RILP, PRPF8, TLCD2, WDR81, SERPINF2, SERPINF1, SMYD4, RPA1, RTN4RL1, DPH1, OVCA2, HIC1, SMG6, SRR, TSR1, SGSM2, MNT, METTL16, PAFAH1B1, CLUH, CCDC92B, RAP1GAP2, OR1D5, OR1D2, OR1E3, OR1G1, OR1A2, OR1A1, OR3A2, OR3A1, OR1R1P, OR1E1, OR3A3, OR1E2, SPATA22, ASPA, TRPV3, TRPV1, SHPK, CTNS, TAX1BP3, EMC6, P2RX5, ITGAE, HASPIN, NCBP3, CAMKK1, P2RX1, ATP2A3, ZZEF1, CYB5D2, ANKFY1, UBE2G1, SPNS3, SPNS2, MYBBP1A, GGT6, SMTNL2, ALOX15, PELP1. | Genomic Position: NC_000017.10:g.pTer_(4579802_?)del (GRCh37)  Cytogenetic Band: 17p13.3p13.2  Type: Deletion Zygosity: Heterozygous Inheritance: Unknown | The variant is not observed in the gnomAD SVs v2.1.1 dataset | | None | | There are multiple similarly affected individuals reported with similar likely pathogenic copy-number-loss overlapping this region ( ClinVar ID: VCV000057265.1, VCV002685590.1). | | None | Other pathogenic variant of the same consequence have been reported in this region(s). | Autosomal dominant 'Miller-Dieker lissencephaly syndrome (OMIM: 247200) | Not performed as the variant was considered high-quality | | Pathogenic | |  | |  |
| 26 | POSITIVE | There is no known syndrome associated with deletion at this region yet | DEFB125, DEFB126, DEFB127, DEFB128, DEFB129, DEFB132, C20orf96, ZCCHC3, SOX12, NRSN2, TRIB3, RBCK1, TBC1D20, CSNK2A1. | Genomic Position: NC_000020.10:g.(?_68351)_(489195_?)del (GRCh37) Cytogenetic Band: 20pterp13 (minimum size: 420.8Kb) Type: Deletion Zygosity: Heterozygous Inheritance: Unknown | The variant is not observed in the gnomAD SVs v2.1.1 dataset | | The heterozygous deletion NC_000020.10:g.(?_68351)_(489195_?) at 20pterp13 spans across 14 genes | | None | | None | Although there is no known syndrome associated with deletion at this region yet, there are multiple similarly affected individuals reported (PMID: 31087544) | There is no known syndrome associated with deletion at this region yet | Not performed as the variant was considered high-quality | | Pathogenic | |  | |  |
